# USP1 inhibits influenza A and B virus replication in MDCK cells by mediating RIG-I deubiquitination

**DOI:** 10.1007/s00018-025-05733-6

**Published:** 2025-05-14

**Authors:** Yuejiao Liao, Siya Wang, Tian Tang, Chengfang Li, Chenhao Yang, Liyuan Ma, Jin Ye, Jiamin Wang, Di Yang, Zilin Qiao, Zhongren Ma, Zhenbin Liu

**Affiliations:** 1 Engineering Research Center of Key Technology and Industrialization of Cell-Based Vaccine, Ministry of Education, Northwest Minzu University, Lanzhou, 730030 China; 2 Gansu Tech Innovation Center of Animal Cell, Biomedical Research Center, Northwest Minzu University, Lanzhou, 730030 China; 3 Key Laboratory of Biotechnology & Bioengineering of State Ethnic Affairs Commission, Biomedical Research Center, Northwest Minzu University, Lanzhou, 730030 China; 4 Life Science and Engineering College of Northwest Minzu University, Lanzhou, 730030 China; 5 Department of Experiment & Teaching, Northwest Minzu University, Lanzhou, 730030 China

**Keywords:** Innate immunity, Influenza virus, USP1, RIG-I, MDCK cells, Vaccine production

## Abstract

The post-translational modification and stability regulation of RIG-I play critical roles in promoting IFN-I production and maintaining immune homeostasis. In this study, we found that ubiquitin-specific peptidase 1 (USP1) promotes RIG-I protein stability through deubiquitination, which in turn enhances antiviral immunity through the production of inflammatory cytokines, and inhibits the replication of influenza virus in MDCK cells. In contrast, USP1 knockdown inhibited the deubiquitination of RIG-I, decreased the RIG-I protein level, and significantly increased the influenza virus titer. Meanwhile, inhibition of USP1 expression did not have a significant effect on the proliferation of MDCK cells, suggesting that USP1 could be used as a target gene to establish a vaccine-producing MDCK cell line. The above results provide a more comprehensive understanding of the function of USP1 and the antiviral response mechanism, and provide a theoretical and methodological basis for the screening of target genes for the artificial establishment of high-yield MDCK cell lines for vaccine production.

**Supplementary Information:**

The online version contains supplementary material available at 10.1007/s00018-025-05733-6.

## Introduction

Influenza is a common respiratory infectious disease caused by influenza virus infection, and influenza pandemics pose a great threat to global public health every year. Currently, influenza vaccination is the most effective means to prevent and control influenza virus infection [1]. Madin-Darby Canine Kidney (MDCK) cells are highly sensitive to influenza viruses; moreover, the advantages of these cells—high infection efficiency, low operating cost, rapid proliferation, and low susceptibility to mutation—have led to their worldwide recognition as one of the main cell lines for use in influenza vaccine production [2]. Meanwhile, MDCK cells are also commonly used as host cells to study the regulatory mechanism of influenza virus proliferation. Additionally, recent research on the construction of vaccine-producing cell lines has increasingly focused on the use of gene editing technology to modify host target genes that significantly affect viral replication. For example, interferon regulatory factor 7 (IRF7) knockdown in MDCK cells produces high titers of influenza virus, and signal transducer and activator of transcription 1 (STAT1) knockdown reduces the titer of progeny viruses and the synthesis of viral proteins in cells infected with influenza A virus H1 N1 [3, 4]. Therefore, the construction of high-yield influenza vaccine cell lines genetically engineered to target host antiviral proteins is an important means to effectively increase the level of viral replication in cells and improve vaccine production efficiency. This improvement in vaccine production also requires a more comprehensive understanding of the regulatory mechanisms of viral infection and replication within cell lines.

The ubiquitin–proteasome system (UPS) and the mechanism of ubiquitination are key processes in numerous pathways, including the innate immune response, autophagy, cancer therapy [5–9]. The entire ubiquitination system—ubiquitin, proteasomes, and enzymes including members of the ubiquitin-specific peptidase (USP) family, a family of proteins with deubiquitinating enzyme activity—is involved in the regulation of a wide range of intracellular biological processes by regulating the localization, stability, and function of proteins that affect cell growth, differentiation, apoptosis, autophagy, and DNA damage repair [10–14]. Some evidence suggests that some members of this family are involved in the regulation of viral responses in host cells by stabilizing the structure of substrate proteins and regulating various signaling pathways [15–17]. For example, USP25 protects tumor necrosis factor receptor-associate factor 3 (TRAF3) and TRAF6—which are involved in the Toll-like receptor 4 (TLR4)- and interleukin-17 (IL-17)-mediated signaling pathways—from degradation by abrogating the K48-linked ubiquitination of TRAF3 and the K63-linked ubiquitination of TRAF6, which thereby promotes virus-induced type I interferon (IFN-I) and proinflammatory factors that positively regulate the innate antiviral immune response [18–20].After DNA virus infection, USP44 is recruited to the interferon regulatory factor 3 (IRF3) activation mediator (MITA, STING) and removes the K48-linked polyubiquitinated portion from MITA at K236, thereby preventing the proteasome-mediated degradation of MITA [17]. Another study demonstrated that USP2b can cleave the K63-linked polyubiquitin chain from TANK-binding kinase 1 (TBK1), thereby inhibiting its kinase activity, which is consistent with the inhibitory effect of USP2b on TBK1 activation; moreover USP2b knockdown markedly inhibits vesicular stomatitis virus (VSV) replication [21].

USP1, a member of the USP family, is a deubiquitinating enzyme (DUB) with His and Cys structural domains, and it is involved in the regulation of various signaling pathways by mediating the deubiquitination of substrate proteins [22–24]. USP1 has been shown to mediate the deubiquitination of TBK1 and stabilize its expression by binding to USP1-associated factor 1 (UAF1) to form a complex that enhances the secretion of IFN-β and inhibits the proliferation of VSV in human embryonic kidney (HEK)−293 cells and mouse macrophages [25]. These findings lead to several questions that remain unknown. Does the USP1 gene have an antiviral function in the regulation of the influenza virus response in MDCK cells, and could it therefore serve as an effective target gene to promote influenza vaccine yield? Is USP1 involved in the regulation of influenza virus replication in MDCK cells by the same molecular mechanism as in human cells?

In this study, we investigated the function and mechanism of USP1 on influenza A and B virus replication in MDCK cells. To understand the influence of USP1 on influenza virus replication in MDCK cells, we overexpressed or knocked down the USP1 gene in MDCK cells and analyzed its interactions with other molecules and the effects on the signaling pathways involved in the influenza virus response. We then further investigated the mechanism of influenza virus replication in MDCK cells. The results of this study create a more comprehensive understanding of the molecular regulatory mechanism of the host cell innate immune response against influenza virus, while simultaneously laying a theoretical and methodological foundation for the artificial establishment of high-yield MDCK cell lines for influenza vaccine production, with promising possibilities for application.

## Material and method

### Cells and virus

MDCK adherent cells (ATCC,#CCL-34), A549 cells, HEK-293 cells were kindly provided by Gansu Tech Innovation Center of Animal Cell, China.Influenza A/Puerto Rico/8/34 (A/PR/8/34) H1 N1 virus, A/Texas/50/2012 (H3 N2) NYMCX- 223 A, B/Colorado/06/2017-like virus B (Victoria lineage), B/Phuket/3073/2013-like virus B (Yamagata/16/88 lineage) were provided by the Vaccine Research Laboratory II of Wuhan Institute of Biological Products Co.,Ltd and kept at −80℃. MDCK and HEK-293 cells were cultured in DMEM medium supplemented with 10% NBS serum, A549 cells were cultured in F12 medium supplemented with 10% NBS serum, and all cells were cultured at 37℃ with 5% CO_2_.

### Transfection of lentiviral

Carrier were provided by Shanghai JiKai Biotechnology Co. Wildtype MDCK cells with good growth status were inoculated into 24-well plates at 1 × 10^5^ cells/well, and the cells were digested and counted when they had grown to 50–70%, and the lentiviral fluids of USP1 RNAi (102726–1, 102727–1, 102728–1), the lentiviral fluids of USP1 overexpression and its control cytoviral fluid CON313 (purchased from Shanghai Jikai Genomics Science and Technology Co. Ltd.) was inoculated into MDCK cells at MOI = 80, and then replaced by DMEM medium containing 10% NBS 12 h later. After 12 h, the cells were replaced with DMEM medium containing 10% NBS and incubated at 37℃ in 5% CO_2_ incubator for 48 h. The fluorescence expression was then observed under a fluorescence microscope and the cells were subcultured. After cell adherence, DMEM supplemented with 4% NBS was added and 4 µg/mL puromycin was used to screen the cell resistance. After co-culture with puromycin for 5 generations, stable cell lines with USP1 overexpression or knockdown were obtained.

### Influenza virus infectivity assays

MDCK adherent cells, HEK-293 cells, A549 cells, overexpression control (LV-control) cells, overexpression (LV-USP1) cells, knockdown control (sh-control) cells, and knockdown (sh-usp1) cells were inoculated into 6-well plates with a well of 7 × 10^5^/well and then washed three times with PBS. The cells were washed three times with PBS, and then infected with H1 N1, H3 N2, BY, and BV (MOI = 0.01) influenza viruses diluted in serum-free DMEM containing 2 μg/mL TPCK trypsin (trypsin was not added to HEK-293 cells), and then the virus supernatant was discarded. One-hour post-infection, the virus supernatant was discarded, and 3 ml serum-free DMEM medium with 2 μg/ml TPCK pancreatin was cultured at 34 ℃ with 5% CO_2_.Virus supernatant, RNA, and protein samples were collected at 12, 24, 36, and 48 h for further analysis.

### Western blot (Wb)

Each cell sample was combined with a 200 µL PMSF/RIPA buffer mixture (PMSF:RIPA = 1:100) and proteins were quantified using the BCA protein assay. To denature the proteins, 75 µL of each protein sample were added to 20 µL 5 × loading buffer and placed in a water bath at 100 ℃ for 10 min. The denatured proteins were separated using SDS-PAGE (120 V, 120 min) with 7.5–15% precast gels (Bio-Rad, Hercules, CA, USA) and transferred to membranes (220 mA, 90 min). The membranes were blocked with 5% skim milk powder and sealed at room temperature for 2 h, incubated with the primary antibody (1:1000 dilution) at room temperature for 2 h, washed with TBST, incubated with secondary HRP-labeled goat anti-mouse IgG or goat anti-rabbit IgG antibody (1:5000 dilution) at room temperature for 1 h, washed with TBST, and visualized after ECL color development (Sinsitech, MiniChemi 610, China) to determine relative protein expression.

RIPA lysis buffer (PC101), PMSF (GRF101), and a BCA Protein Quantitative Kit (ZJ101) were purchased from Shanghai Yamei Biomedical Technology Co., Ltd.; SDS-PAGE loading buffer, 5 × (P1040), and a WB gel making kit (A1010) were purchased from Beijing Solarbio Science & Technology Co., Ltd.; low molecular weight poly(I:C) is from InvivoGen Co., Ltd; PBS buffer, DMEM, 1640 medium were purchased from Cellmax Technology (Beijing) Co., Ltd; Newborn bovine serum/fetal bovine serum were purchased from Lanzhou Minhai Bioengineering Co., Ltd; Canine IFN-α from Zhongkebaike Biotechnology Co., Ltd; Anti-flag murine monoclonal antibody (cat: T0003) was purchased from affinity; Anti IBV NP rabbit monoclonal antibody (cat: B017) was purchased from Abcam; Goat anti-mouse IgG (cat: SA00001-1)/Goat anti-rabbit IgG (cat: SA00001-2)/anti USP1 mouse polyclonal antibody (cat: SA00013-3) were purchased from Proteintech; resist β-actin murine monoclonal antibody (cat:66,009) was purchased from CST.

### Realtime-qPCR

Total RNA was extracted by Trizol reagent, and cDNA was synthesized by reverse transcription; SYBR green fluorescent quantitative PCR was used to detect gene expression changes. The PCR reaction condition is: pre-denaturation at 95 ℃ for 15 min; denatured at 95 ℃ for 10 s, annealed at 60 ℃ for 20 s, and extended at 72 ℃ for 30 s, a total of 40 cycles. The fusion curve was analyzed after amplification. GAPDH was used as the internal reference gene, and the relative transcription level of the gene was calculated using the 2^−△△Ct^ method.

### TCID50

After adding 1 × 10^4^ MDCK cells to a 96-well cell plate, the plate was cultured at 37 ℃ and 5% CO_2_ for 24 h. We discarded the culture medium, washed the cells twice with PBS, obtained the viral supernatant to be tested in the 96-well cell plate, and used the virus maintenance solution (1 μg/ml trypsin DMEM) for logarithmic dilution. The dilution degree was 10^–1^ ~ 10^–11^ in turn; different dilutions of virus solution were added to columns 1–11 (100 μl/well) of the 96 well cell plate; 1 μg/ml trypsin DMEM (100 μl/well) was added to column 12 as the negative control. The cells were incubated in a 5% CO_2_ incubator at 34 ℃ for 72 h. Added 100 μL crystal violet staining for 10 ~ 20 min. The results were judged according to the cytopathic effect (CPE). If > 50% of the area at the bottom of the hole was stained purple, the hole was considered negative.

### Co-immunoprecipitation

Cell samples with or without IAV infection were mixed with 1 mL of RIPA buffer (PMSF:RIPA = 1:100) for protein quantification using BCA quantification method. Pierce Anti-c-Myc Magnetic Beads 25ul (Thermo Fisher Scientific) was taken for pretreatment and added to the protein samples for incubation, and after two washes, 100ul of 1 × loading buffer was added, and then incubated at 100 °C for 10 min to denature the proteins. And then the Wb experiment was performed.

### Proteomic analyses

For proteomic analysis, MDCK cells were infected with H3 N2 influenza virus (MOI = 1) for 12 h. Protein extraction was performed by adding 50 μL SDT lysis buffer to 100 μL aliquots of each sample, followed by sonication and 10-min boiling. After centrifugation (16,000 × g, 4 °C), protein concentration was determined using the BCA assay. Samples were then diluted fivefold, mixed with 5 × loading buffer, boiled for 5 min, and resolved on 8–16% gradient SDS-PAGE gels. Excised gel strips were subjected to in-gel trypsin digestion and peptide desalting, using iTRAQ quantitative proteomics technology. Peptides were fractionated using a High pH Reversed-Phase Peptide Fractionation Kit (Thermo Fisher Scientific), yielding 15 fractions that were vacuum-dried and reconstituted in 0.1% formic acid. Afterwards, the peptides were separated by nano-scale flow rate HPLC system Easy nLC and analysed by liquid chromatography-tandem mass spectrometry (LC–MS) using Q- Exactive Plus mass spectrometer (Thermo Fisher Scientific). Chromatographic separation used a 75 μm × 25 cm analytical column with a 120-min gradient. Mass spectrometry parameters included: MS1: Resolution 70,000 (@ m/z 200), AGC target 1e6, maximum injection time 50 ms. MS2: Resolution 17,500 (@ m/z 200), AGC target 1e5, maximum injection time 50 ms, HCD activation with 35% normalized collision energy, Isolation window: 1.6 Th.

Raw data were processed using MaxQuant (v1.6.0.16) against the Canis lupus familiaris UniProt database (release 20180207 containing 29,580 entries, downloaded from https://www.uniprot.org).

### Statistical analysis

Data were analyzed with GraphPad Prism 5.0 using one-way ANOVA or Student’s t-test, and analyses are presented as the mean ± standard deviation (SD) of three independent experiments. A p-value < 0.05 was considered statistically significant and marked as “*” while *p* < 0.01, and p < 0.001, marked as “**” and “***” respectively, corresponding to: *p < 0.05; **p < 0.01; ***p < 0.001.

## Results

### Influenza virus infection induces intracellular USP1 expression

**Fig. 1:**
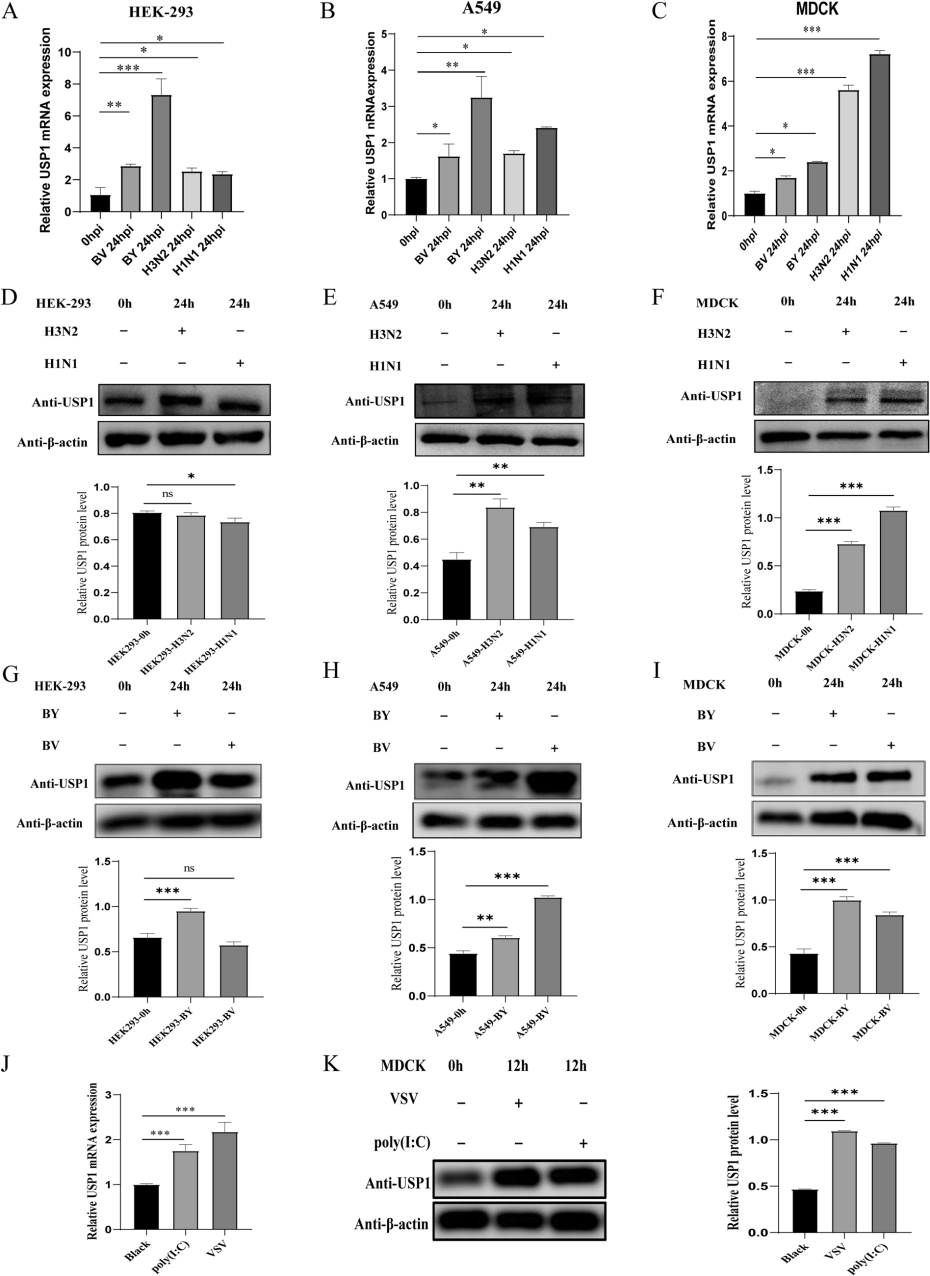
Influenza virus infection induces upregulation of USP1 expression. (**A**–**C**) Changes in USP1 mRNA expression were analyzed in HEK-293, A549, and MDCK cells after 24 h of influenza virus infection using RT-qPCR. (**D**–**I**) Changes in USP1 protein expression were analyzed in HEK-293, A549, and MDCK cells after influenza virus infection using Wb. (**J**, **K**) MDCK cells were stimulated with VSV (MOI = 0.001) or 5 μmol/mL low molecular weight poly(I:C) and changes in USP1 (**J**) mRNA and (**K**) protein expression were detected 12 h post-stimulation. * *p* < 0.05; ** *p* < 0.01; *** *p* < 0.001

The ability of USP1 to regulate influenza virus replication has not been reported. Using proteomics analysis, we found that the protein expression level of USP1 was significantly up-regulated in MDCK cells infected with influenza A virus H3 N2 for 12 h (Fig. S1 and Table S1). We further examined the changes in USP1 mRNA and protein levels after infection with different influenza viruses (MOI = 0.01) in MDCK, A549, and HEK-293 cells using RT-qPCR and Wb techniques, respectively. The results showed that the expression of USP1 mRNA levels was significantly up-regulated 24 h after infection with all four influenza viruses-A/PR/8/34 (H1 N1), A/Texas/50/2012 (H3 N2), B/Colorado/06/2017 (BV), and B/Phuket/3073/2013 (BY) (Fig. 1A-C). At the protein level, the expression of USP1 protein levels was significantly upregulated in all four influenza viruses infected with USP1 in A549 cells and MDCK cells, whereas the expression of USP1 protein levels was upregulated in HEK293 cells only with BY infection (Fig. 1D-I). Although there were slight differences between different cells and viruses, which may be due to cell specificity and virulence species, overall influenza virus infection induced USP1 expression in cells, suggesting that USP1 is likely to play a regulatory role in host cell response to influenza virus. In the subsequent experiments, we chose MDCK cells, which are the most sensitive to all four viruses, for the experiments.To explore the signalling pathways that induce USP1 expression, we used 5 μmol/mL of low molecular weight poly(I:C) or vesicular stomatitis virus (VSV) (MOI = 0.001) to stimulate MDCK adherent cells, and found that both stimulants could significantly induce endogenous USP1 mRNA and protein expression in MDCK cells after 12 h (Fig. 1J, K). These findings suggest that influenza virus infection may trigger upregulation of USP1 expression through activation of the RIG-I/MDA5-mediated antiviral signaling pathway.

### Overexpression of USP1 inhibits influenza virus replication in MDCK cells

**Fig. 2:**
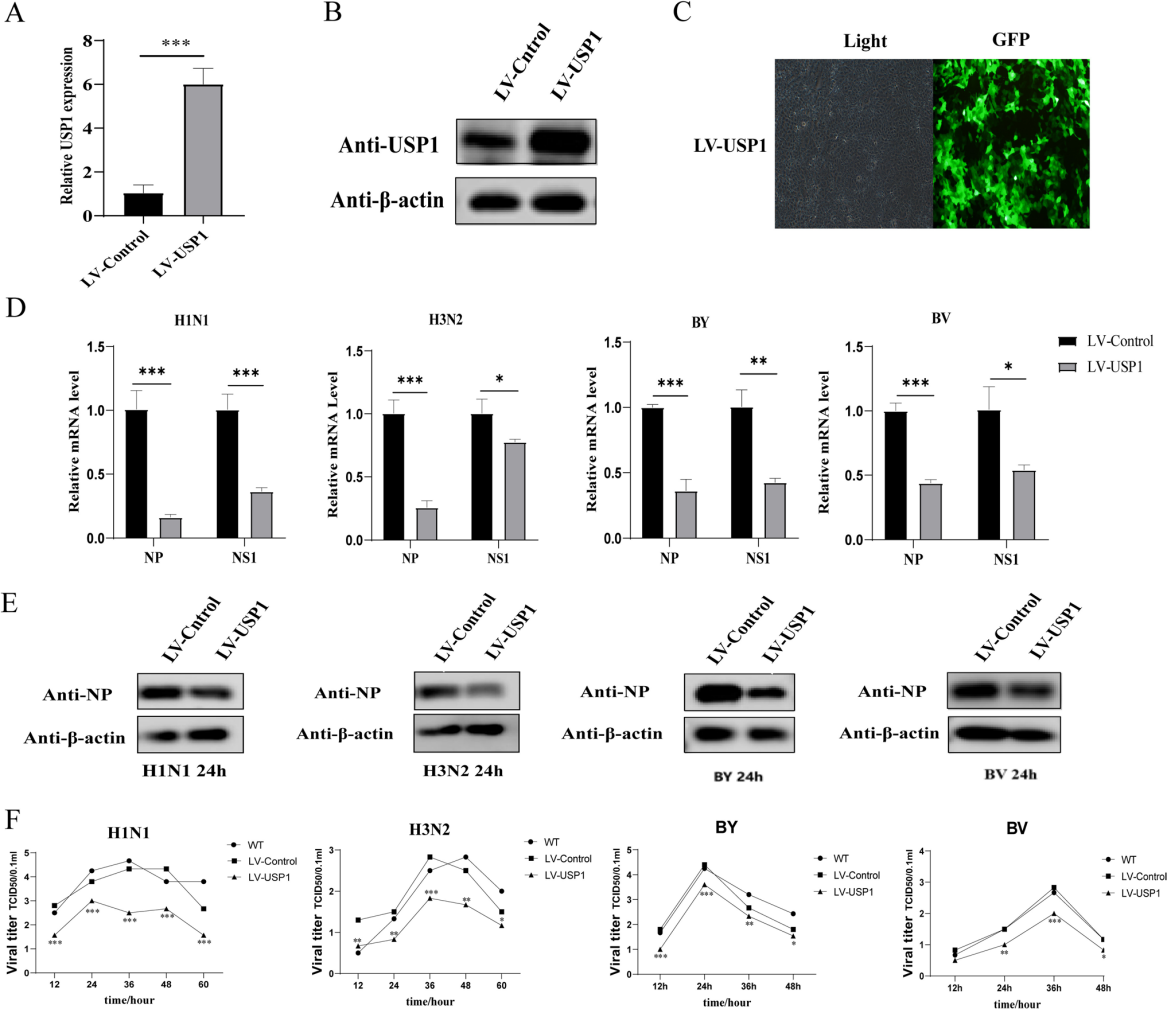
Overexpression of USP1 inhibited influenza virus proliferation. (**A**, **B**) USP1 expression was analyzed in LV-Control and LV-USP1 cells using (**A**) RT-qPCR and (**B**) Wb. (**C**) LV-USP1 labeled with GFP was observed using fluorescence microscopy (40 ×). (**D**) LV-Control and LV-USP1 cells were infected with H1 N1, H3 N2, BY, and BV virus strains, samples were collected 24 h post-infection, and NP and NS1 gene expression levels were measured using RT-qPCR. (**E**) LV-Control and LV-USP1 cells were infected with H1 N1, H3 N2, BY, and BV virus strains, samples were collected 24 h post-infection, and NP protein expression levels were measured using Wb. (**F**) Influenza virus strains H1 N1, H3 N2, BY, and BV were analyzed for viral potency in wild-type (WT), LV-Control, and LV-USP1 cells using the TCID50 method. LV-Control was used as a control. * *p* < 0.05; ** *p* < 0.01; *** *p* < 0.001

To demonstrate that USP1 regulates influenza virus replication in MDCK cells, we transfected lentiviral (LV) USP1 overexpression vectors to increase intracellular USP1 expression. The fluorescence ratio of the cells was observed under fluorescence microscope, and the fluorescence number of the cells was > 90%. Using RT-qPCR and Wb to detect USP1 mRNA and protein expression, respectively, the experimental results showed that both the USP1 mRNA and protein expression levels were significantly upregulated in LV-USP1 cells compared with LV-Control cells (Fig. 2A–C). LV-Control, LV-USP1, and wild-type (WT) cells were infected with four subtypes of influenza strains (H1 N1, H3 N2, BY, BV) at MOI = 0.01, and the supernatant samples were collected at 12 h, 24 h, 36 h, 48 h, and 60 h post-infection for the TCID50 assay. Cell samples were collected 24 h post-infection, and the mRNA levels of influenza virus NP and NS1 gene were measured. The results showed that USP1 knockdown significantly decreased the expression of influenza virus NP and NS1 gene in MDCK cells (Fig. 2D). The protein results were consistent with the mRNA results that USP1 knockdown significantly decreased the expression of influenza virus NP gene in MDCK cells (Fig. 2E). The TCID50 results showed that USP1 overexpression significantly suppressed the replication of influenza A virus H1 N1 and influenza B virus BY in MDCK cells (Fig. 2F).

### Knockdown of USP1 promotes influenza virus replication in MDCK cells

**Fig. 3:**
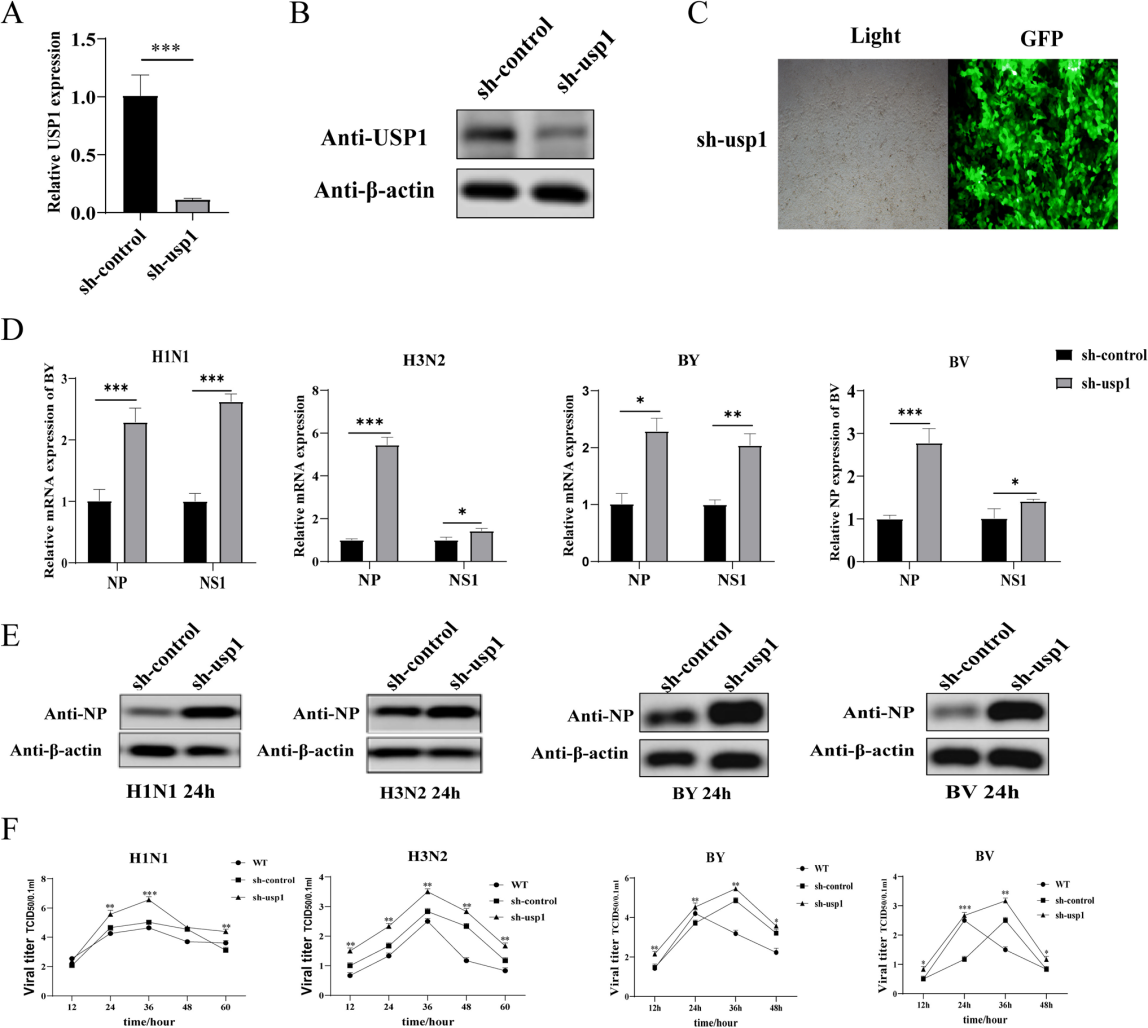
Knockdown of USP1 promoted influenza virus proliferation. (**A**, **B**) USP1 (**A**) gene and (**B**) protein expression was analyzed in sh-control and sh-usp1 cells using RT-qPCR and western blot (Wb), respectively. (**C**) sh-usp1 labeled with GFP was observed using fluorescence microscopy (40 ×). (**D**) sh-control and sh-usp1 cells were infected with H1 N1, H3 N2, BY, and BV virus strains, samples were collected 24 h post-infection, and NP and NS1 gene expression levels were measured using RT-qPCR. (**E**) sh-control and sh-usp1 cells were infected with H1 N1, H3 N2, BY, and BV virus strains, samples were collected 24 h post-infection, and NP protein expression levels were measured using Wb. (**F**) Influenza virus strains H1 N1, H3 N2, BY, and BV were analyzed for viral potency in wild-type (WT), sh-control, and sh-usp1 cells using the TCID50 method. sh-control is the control. * *p* < 0.05; ** *p* < 0.01; *** *p* < 0.001

**Fig. 4:**
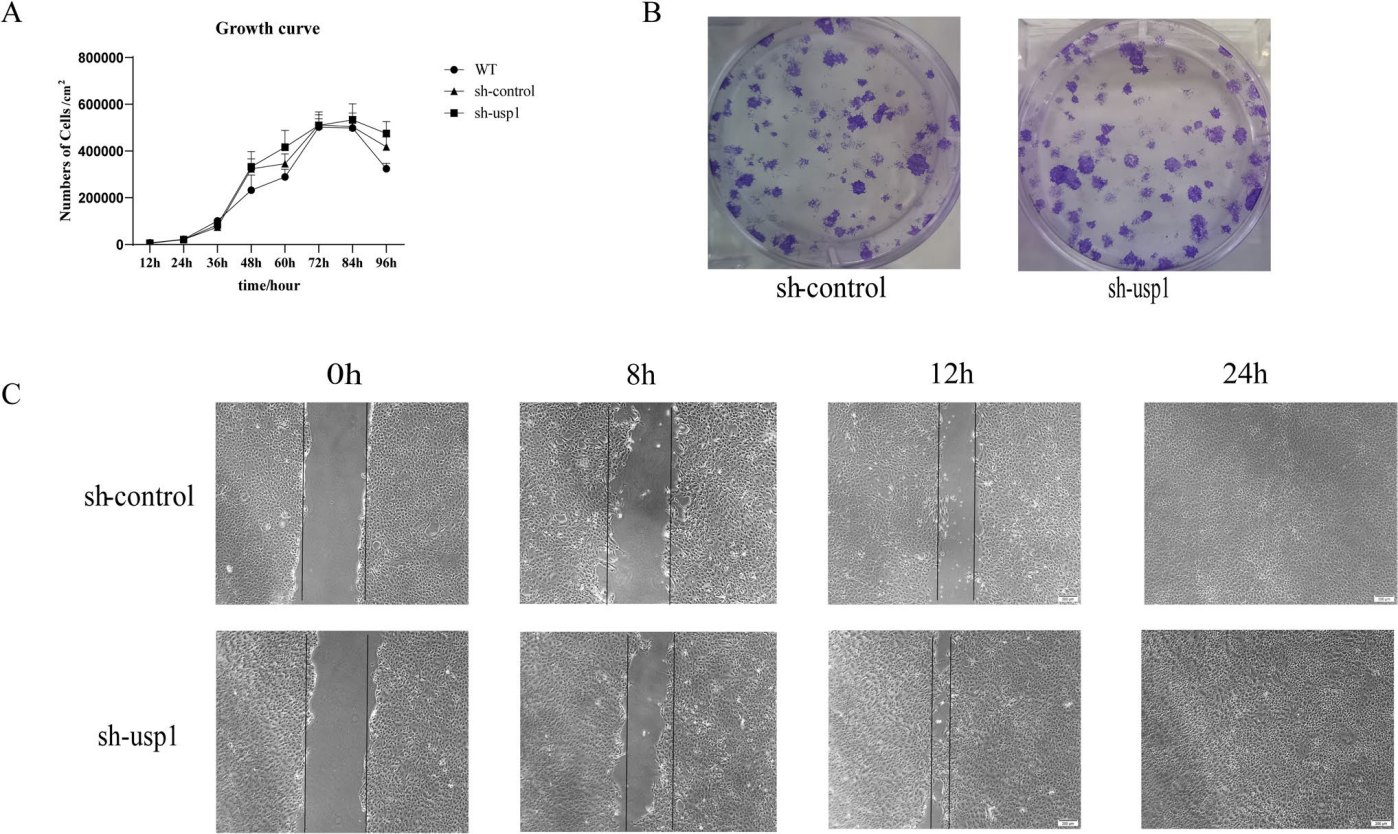
Knockdown of USP1 does not affect MDCK cell proliferation. (**A**) Cell proliferation assays were performed on sh-control, sh-usp1, and wild-type (WT) cells using cell digestion and counting. (**B**) The cell clone formation assay was performed on sh-control and sh-usp1 cells at 500 cells/well. (**C**) The migration ability of sh-control and sh-usp1 cells was detected using the scratch healing assay

To further investigate the effect of USP1 on influenza virus replication in MDCK cells, we designed a shRNA targeting USP1 mRNA to inhibit intracellular USP1 expression. The fluorescence ratio of the cells was observed under fluorescence microscope, and the fluorescence number of the cells was > 90%. The mRNA and protein expression of USP1 was detected using RT-qPCR and Wb, respectively, which showed that both the mRNA and protein levels of USP1 were significantly down-regulated in the sh-usp1 cells compared with the sh-control cells (Fig. 3A–C). After infecting the sh-control, sh-usp1, and WT cells with four subtypes of influenza strains (H1 N1, H3 N2, BY, BV) at MOI = 0.01, samples of the supernatants were collected at 12 h, 24 h, 36 h, 48 h, and 60 h post-infection for the TCID50 assay. Cell samples were collected 24 h post-infection, and the mRNA levels of influenza virus NP and NS1 gene were measured. The results showed that USP1 knockdown significantly promoted the expression of influenza virus NP and NS1 gene in MDCK cells (Fig. 3D). The protein results were consistent with the mRNA results that USP1 knockdown significantly promoted the expression of influenza virus NP gene in MDCK cells (Fig. 3E). The TCID50 results showed that USP1 knockdown significantly promoted the replication of influenza A virus H1 N1 and influenza B virus BY in MDCK cells (Fig. 3F). The value-added efficiency of the sh-usp1 cells was determined from trypsin digestion and counting, which showed no significant difference in sh-usp1 cell proliferation relative to control cells, but higher cell migration ability (Fig. 4A, C). Moreover, the plate clone formation assay showed there was no significant difference in the clone formation rate of sh-usp1 cells relative to control cells (Fig. 4B). The above results indicated that USP1 knockdown promoted influenza virus replication in MDCK cells without affecting MDCK cell proliferation.

### USP1 positively increases RIG-I protein levels

**Fig. 5:**
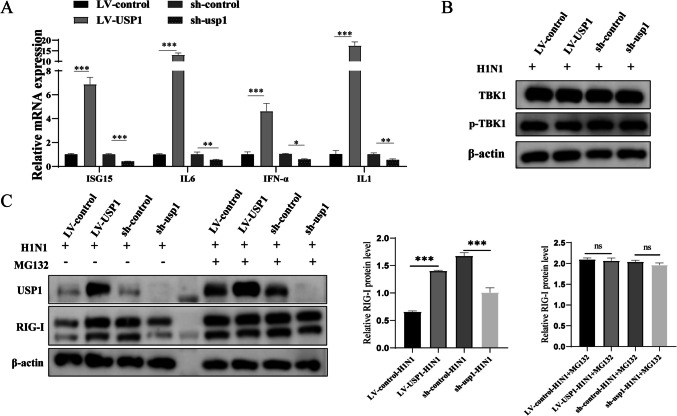
USP1 positively increases RIG-I protein levels. (**A**) LV-control, LV-USP1, sh-control, and sh-usp1 cells were stimulated with 5 μmol/mL low molecular weight poly(I:C) for 24 h. RNA samples were collected, and mRNA expression of key factors downstream of the RIG-I/MDA5 signaling pathway was analyzed in each group using RT-qPCR. (**B**) Changes in TBK1 and p-TBK1 protein expression were analyzed after H1 N1 infection of LV-control, LV-USP1, sh-control, and sh-usp1 cells using Wb. (**C**) Changes in RIG-I protein expression following H1 N1 infection of LV-control, LV-USP1, sh-control and sh-usp1 cells treated with or without MG132 were analysed using Wb. * *p* < 0.05; ** *p* < 0.01; *** *p* < 0.001

To identify the innate immune signalling pathways involved in USP1, after low molecular weight poly(I:C) stimulation, we found that gene expression downstream of the retinoic acid-inducible gene I (RIG-I) receptor-mediated signaling pathway was affected by USP1. The results demonstrated that the expression levels of IFN, antiviral interferon-stimulated genes (ISGs), and downstream signaling factors of RIG-I/MDA5 (including IL-1 and IL-6) were significantly upregulated in LV-USP1 cells but downregulated in sh-USP1 cells (Fig. 5A). This result suggests that USP1 may regulate influenza virus replication by modulating RIG-I signaling and inducing NF-κB activation in MDCK cells, which in turn may mediate the activation of IFN-I and the subsequent production and release of pro-inflammatory cytokines. USP1 has been previously shown in mouse macrophages and HEK-293 cells to affect the stability of TBK1 through deubiquitination and thus regulate VSV replication. Therefore, we tested whether USP1 overexpression or knockdown could significantly affect the TBK1 protein level in MDCK cells. The results showed that both USP1 overexpression and knockdown in MDCK cells had no significant effect on the protein level of TBK1 and phosphorylated TBK1 (p-TBK1), suggesting that different cell types may involve different signaling pathways in the mechanism by which USP1 affects viral replication (Fig. 5B). In addition, we tested whether USP1 overexpression or knockdown could significantly affect the RIG-I protein level in MDCK cells. The results showed that the RIG-I protein expression level was significantly elevated after viral infection in LV-USP1 cells, but significantly decreased in sh-usp1 cells, however, there was no significant change in the protein level expression of RIG-I after MG132 treatment (Fig. 5C). The above results suggest that RIG-I is a key reciprocal target gene that may be involved in USP1-mediated inhibition of influenza virus replication in MDCK cells, and that USP1 may stabilise the protein expression of RIG-I by deubiquitination.

### USP1 interacts with RIG-I and stabilizes RIG-I expression by deubiquitination in MDCK cells

To elucidate the molecular mechanism underlying USP1-mediated regulation of influenza virus replication in MDCK cells, we identified the USP1 interacting partners during infection with the H1 N1 virus. Following immunoprecipitation of 3 × FLAG-tagged USP1, mass spectrometry analysis identified sixteen high-confidence interacting partners (Table S2). Among them, HSPA8, RIG-I, and VCP proteins have been shown to be associated with host antiviral resistance. Together with the previous experiments, we determined that RIG-I may be the target protein of USP1 for its antiviral function.

**Fig. 6:**
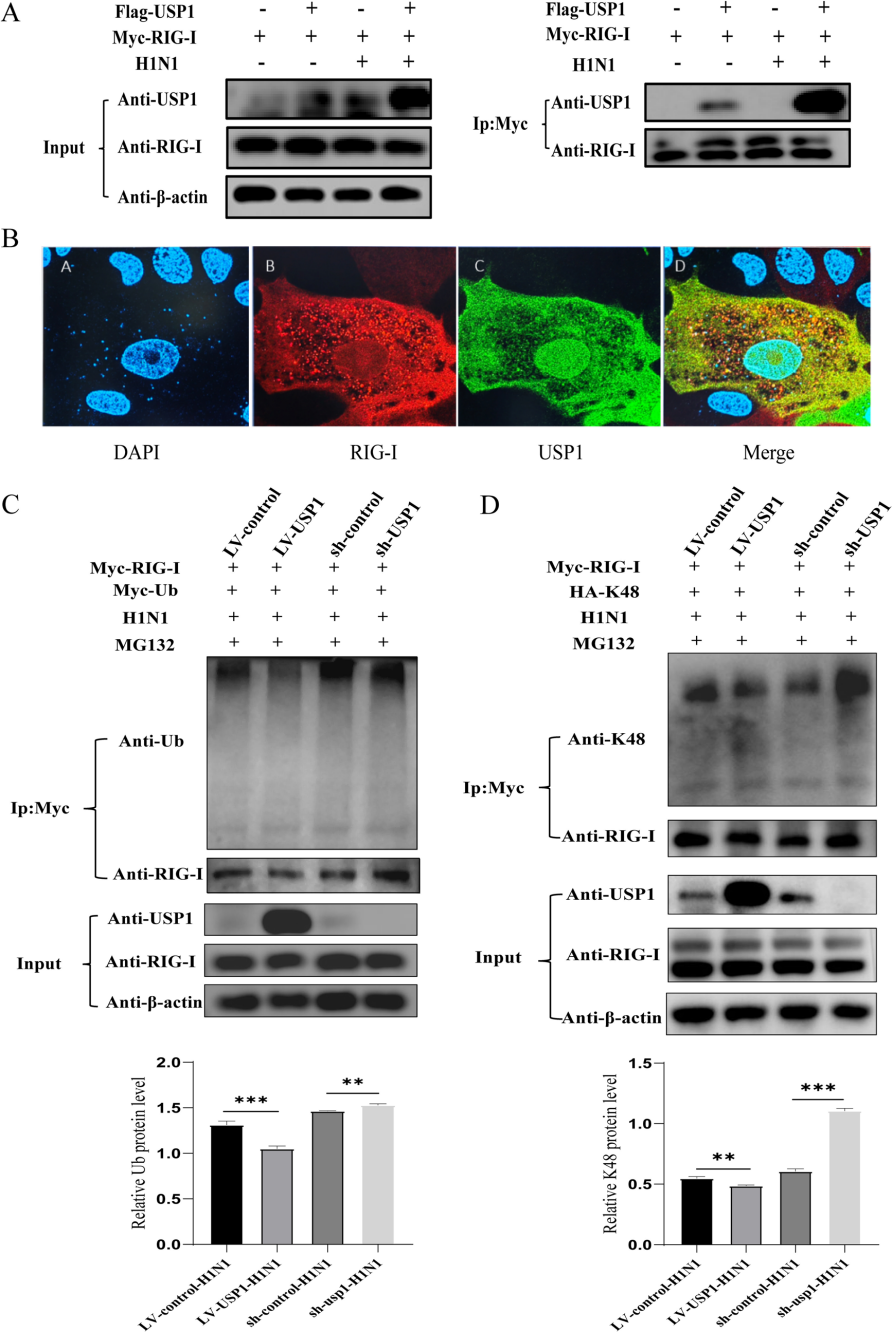
USP1 removes K48-linked ubiquitination of RIG-1 and promotes its stabilization. (**A**) Myc-tagged RIG-I was pulled down using the Co-IP method before and after H1 N1 infection and subjected to Wb analysis. (**B**) A fluorescence co-localization assay was performed to detect the intracellular location of RIG-I and USP1 in MDCK cells using confocal microscopy. (**C**) Myc-tagged RIG-I was pulled down using the IP method after H1 N1 infection. Levels of ubiquitinated (Ub) RIG-1 were measured in LV-control, LV-USP1, sh-usp1, and sh-control cells using Wb. (**D**) Myc-tagged RIG-I was pulled down using the IP method after H1 N1 infection. Levels of K48 ubiquitinated RIG-1 were measured in LV-control, LV-USP1, sh-usp1, and sh-control cells using Wb. * *p* < 0.05; ** *p* < 0.01; *** *p* < 0.001

To determine the interactions between RIG-I and USP1, we performed a Co-IP assay to pull down the Flag-tagged USP1 protein in LV-USP1 cells followed by Wb, which showed that exogenous USP1 could interact with endogenous RIG-I (Fig. S2A).Then, we performed a Co-IP assay to pull down the Myc-tagged RIG-I protein in LV-USP1 cells followed by Wb, which showed that exogenous USP1 could interact with exogenous RIG-I (Fig. 6A). Next, we performed fluorescence co-localization experiments on MDCK cells to detect the intracellular location of RIG-I and USP1 using confocal microscopy, which showed that USP1 co-localized with RIG-I in the cells (Fig. 6B). Taken together, these data suggest that USP1 participates in the host IFN antiviral response by interacting with RIG-I.

The primary molecular function of USP1 is to mediate the deubiquitination of substrate proteins; therefore, detecting whether USP1 regulates the ubiquitination of interacting proteins, and whether this ubiquitination affects protein stability, is the key to revealing the regulatory mechanism. RIG-I, an important member of the RIG-I-like receptor (RLR) protein family, is a key pattern recognition receptor (PRR) for detecting influenza viruses and plays an essential role in antiviral immunity. RIG-I recognizes double-stranded RNA (dsRNA) or 5'triphosphate RNA (5'ppp-RNA) produced by RNA viruses replicating in the cytoplasm [26, 27].The post-translational modification and stability of RIG-I is tightly regulated to mediate the production of IFN-I to maintain immune homeostasis. However, the binding of viral RNA to RIG-I causes conformational changes such as ubiquitination and oligomerization. As a deubiquitinating enzyme, USP1 regulates target protein stability through post-translational modification. To investigate whether USP1-mediated regulation of RIG-I requires its deubiquitinating enzyme activity, we performed IP assays using Myc-tagged RIG-I in LV-USP1 cells. Western blot analysis revealed that USP1 overexpression significantly reduced the overall ubiquitination of RIG-I (Fig. 6C). Quantitative assessment demonstrated a 5% decrease in K48-linked ubiquitination in USP1-overexpressing cells, contrasting with a 50% increase in USP1-knockdown cells (Fig. 6D). However, K63-linked ubiquitination remained unaltered regardless of USP1 expression status (Fig. S2B). Before the use of MG132, RIG-I protein levels exhibited a twofold increase in USP1-overexpressing cells and a 60% reduction in knockdown cells (Fig. 5C). These results collectively indicate that USP1 stabilised RIG-I protein expression by inhibiting K48-linked ubiquitination, and that USP1 knockdown led to ubiquitin degradation of RIG-I.

### Functional recovery

**Fig. 7:**
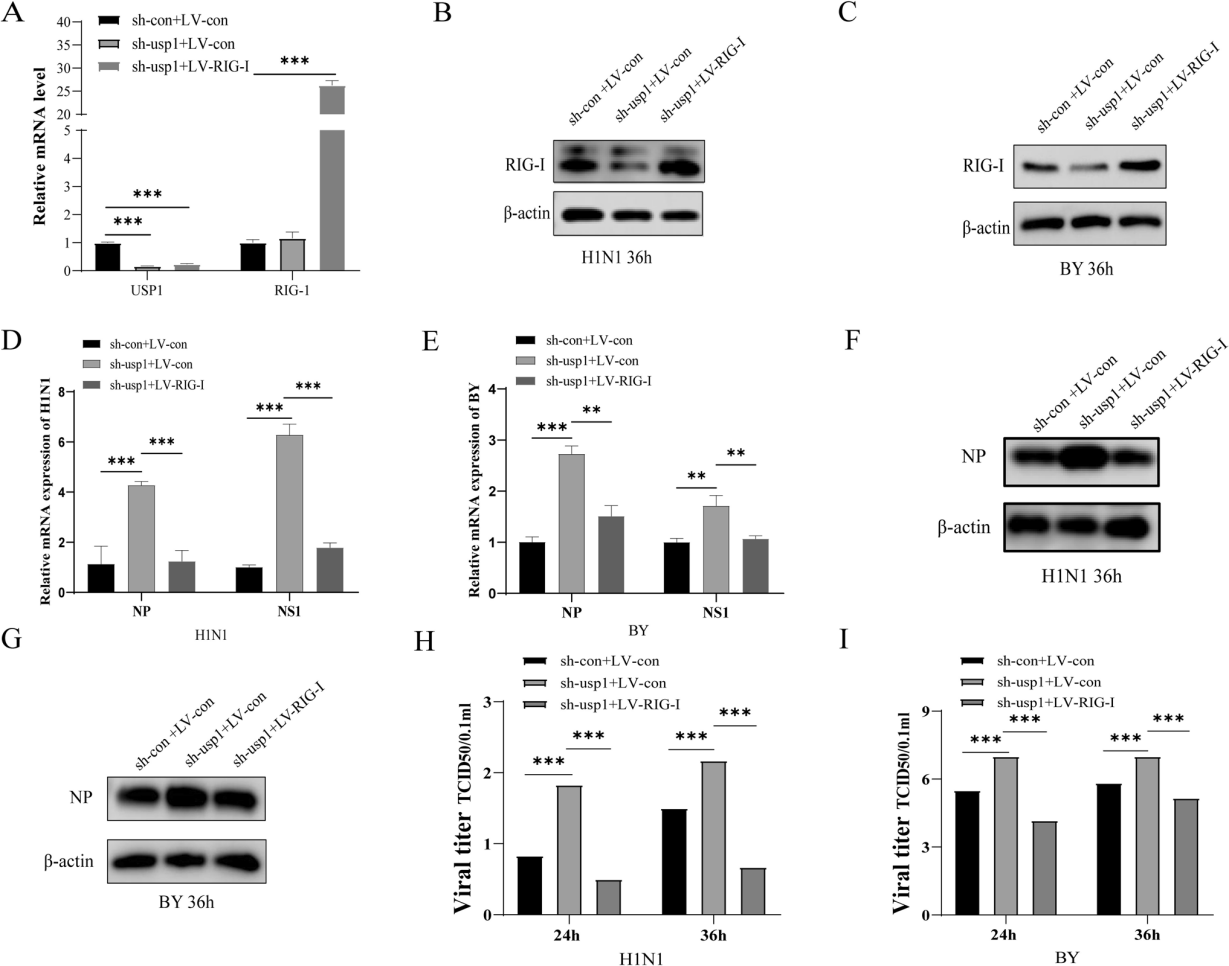
Overexpression of RIG-I positively complements the antiviral effect of USP1. (**A**) USP1 and RIG-I gene expression changes were analyzed in each group of cells using RT-qPCR. Control cells containing empty shRNA and lentiviral vectors (sh-con + LV-con), USP1-knockdown with empty lentiviral vector cells (sh-USP1 + LV-con), USP1-knockdown and RIG-I-overexpressing cells (sh-USP1 + LV-RIG-I). (**B**, **C**) Cells in each group were infected with H1 N1, BY (MOI = 0.01) and cell lysates were collected at 36 h, and RIG-I protein expression was analyzed in cells in each group using Wb. (**D**, **E**) Cells in each group were infected with H1 N1, BY (MOI = 0.01) and cell lysates were collected at 36 h. sh-con + LV-con cells were used as control, and influenza virus NP and NS1 gene expression was analyzed in each group using RT-qPCR. (**F**, **G**) Cells in each group were infected with H1 N1, BY (MOI = 0.01) and cell lysates were collected at 36 h, and influenza virus NP protein expression was analyzed in cells in each group using Wb. (**H**) Viral potency of H1 N1 influenza virus was analyzed after infection in each group of cells using the TCID50 method. (**I**) Viral potency of BY influenza virus was analyzed after infection in each group of cells using the TCID50 method.* *p* < 0.05; ** *p* < 0.01; ***p < 0.001

To further determine whether USP1 affects RIG-I function, we overexpressed a Myc-tagged RIG-I vector in sh-usp1 and sh-control cells and infected them with H1 N1 and BY (MOI = 0.01). Using RT-qPCR to detect USP1 and RIG-I mRNA expression, the experimental results showed that the USP1 mRNA expression levels were significantly down-regulated in sh-usp1 cells compared with sh-control cells, and in the overexpression of RIG-I in sh-usp1 cells, the experimental results showed that the RIG-I mRNA expression levels were significantly upregulated in sh-usp1 cells (Fig. 7A). Following 36 h of infection with either H1 N1 or BY influenza virus (MOI = 0.01), Western blot analysis revealed differential RIG-I protein expression across experimental groups. Specifically, USP1-knockdown cells containing empty lentiviral vector (sh-USP1 + LV-Con) demonstrated significantly reduced RIG-I protein levels (*p* < 0.01). In contrast, the rescue group with combined USP1 knockdown and RIG-I overexpression (sh-USP1 + LV-RIG-I) had higher protein expression of RIG-I (Fig. 7B、C). These findings strongly suggest that USP1 plays a crucial regulatory role in maintaining RIG-I protein stability during influenza virus infection. We found that RIG-I overexpression in sh-usp1 cells significantly inhibited viral NP mRNA and protein expression, and thereby inhibited influenza virus replication (Fig. 7D–I). These results suggest that USP1 enhances the host IFN signaling pathway-mediated antiviral infection response by stabilizing RIG-I protein levels and strengthens the activation and function of the RIG-I-mediated IFN signaling pathway against influenza virus replication.

## Discussion

**Fig. 8:**
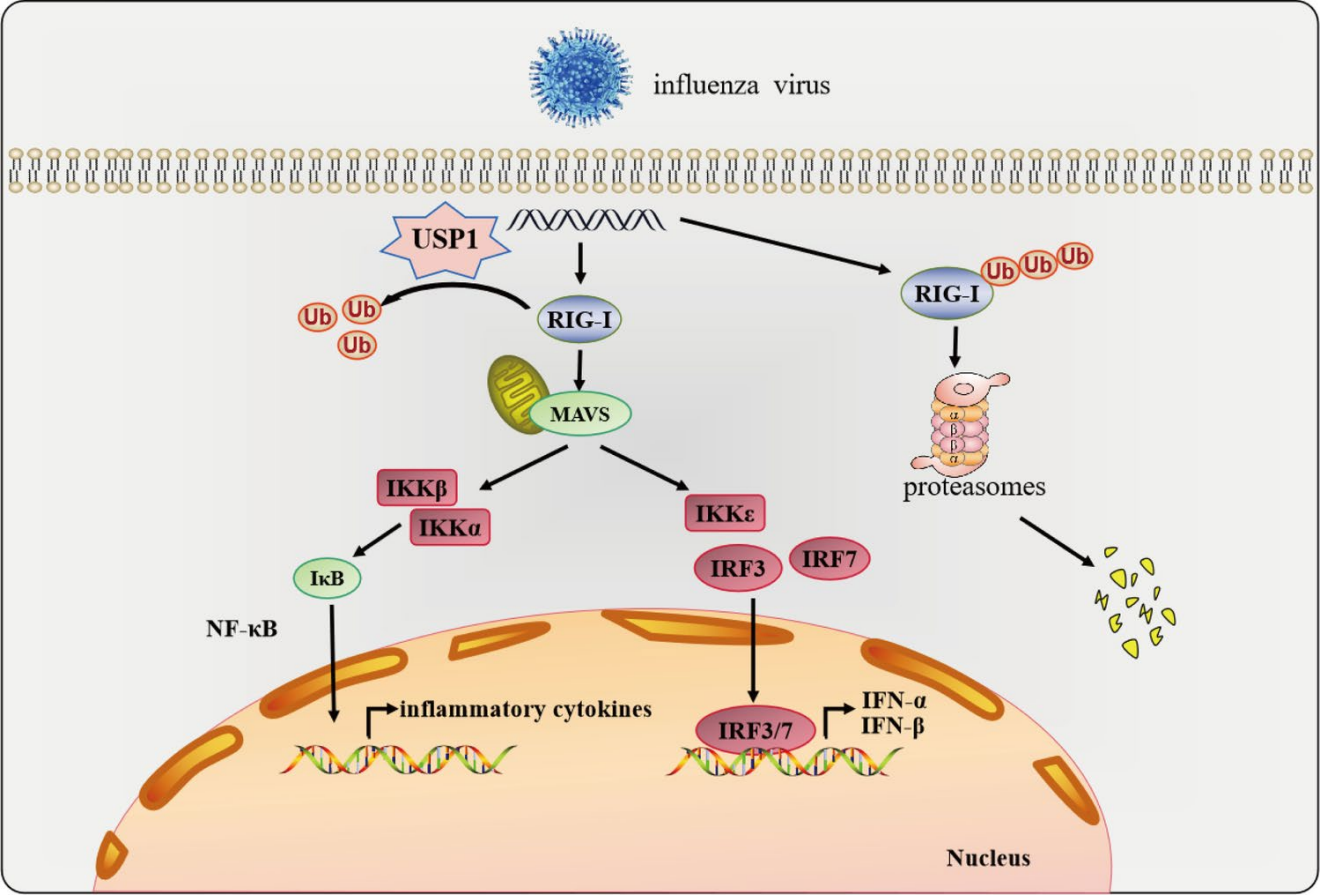
USP1 stabilizes RIG-I protein expression by inhibiting RIG-I ubiquitination

During viral infection, many host cell genes undergo changes in expression and participate in the regulation of viral replication. Most of these genes exert antiviral proliferation functions, while some host genes negatively regulate the antiviral functions and provide favorable conditions for viral proliferation. In our study, we found that influenza virus infection of host cells significantly induced USP1 expression. Using LV expression vectors to construct USP1 overexpression and knockdown MDCK cell lines, we found that USP1 overexpression significantly inhibited the proliferation of influenza virus in MDCK cells, whereas USP1 knockdown significantly promoted intracellular influenza virus replication and viral titers without affecting the proliferation of MDCK cells. In addition, mass spectrometry, Co-IP, immunofluorescence, and ubiquitination analyses revealed that USP1 stabilized RIG-I expression through deubiquitination and promoted activation of the downstream NF-κB signaling pathway, thereby promoting the innate immune response and inhibiting the replication of influenza viruses. Conversely, USP1 knockdown enhanced the ubiquitination of RIG-I and thereby significantly decreased the RIG-I protein levels (Fig. 8). In summary, we reached a preliminary determination that USP1 is an effective target gene for the establishment of high-yield, genetically engineered MDCK cell lines for influenza vaccine production.

The UPS has been reported to play a crucial role in the regulation of host innate and adaptive immunity; a variety of ubiquitin ligases and ubiquitin-binding scaffolding proteins contribute to the positive regulation of antiviral innate immune responses, which is important for host cell resistance to viral infections [28–30].RIG-I is the most important sensor of influenza viruses, and a variety of USP family members can regulate the expression of downstream IFN-I and inflammatory cytokines by modulating the level of RIG-I ubiquitination, and therefore its protein expression. For example, USP3 inhibits IFN-I signaling by deubiquitinating K63-ubiquitinated RLRs [31], USP17 stabilizes RIG-I-induced IFN-I signaling through deubiquitination of K48-linked chains [32], and USP21 inhibits RNA virus-induced RIG-I polyubiquitination and RIG-I-mediated IFN signaling [33]. USP1 is a member of the USP family, which can deubiquitinate a wide range of substrates; furthermore, USP1 and UAF1 form a deubiquitinating enzyme complex that is involved in a variety of biological processes, including DNA repair and tumor pathogenesis [23, 34]. A previous study using the USP1-specific inhibitor ML323 or specific RNAi treatment of mouse peritoneal macrophages found significant inhibition of IFN-β expression, activation of molecules such as IRF3 and STAT1, and TBK1 protein level expression. Using immunoprecipitation and ubiquitination analyses, the USP1-UAF1 complex was found to stabilize TBK1 expression by mediating its deubiquitination and inhibiting its K48-coupled ubiquitination, enhance the TLR3/4- and RIG-I-induced activation of IRF3 and production of IFN-β, and inhibit the vesicularity and proliferation of VSV in HEK-293 cells and mouse macrophages; therefore, the USP1-UAF1 complex could be a potential target for the prevention of viral diseases [25]. Our results also revealed that USP1 inhibited influenza virus replication in MDCK cells by stabilizing RIG-I expression through deubiquitination and promoted the downstream NF-κB signaling pathway without affecting TBK1 protein expression. We speculate that although human USP1 and canine USP1 proteins have very high sequence similarity (Fig. S3), this lack of effect on TBK1 protein expression in MDCK cells may be due to cell-specific variations in the network of interacting proteins, the mechanism of which deserves further exploration and study.

Influenza pandemics are a serious threat to human life and health and socioeconomic development [35]. Vaccines can effectively control the outbreak of influenza in the population; therefore, the timely provision of adequate and effective influenza vaccines is currently the preferred method in many countries to address influenza pandemics. The high safety and mature production technology of inactivated vaccines makes them the most commonly used type of influenza vaccine. MDCK is the main cell line for influenza vaccine production; therefore, focusing on the immune response of this cell to influenza virus infection will help us to understand the mechanism of natural immune signaling against influenza viruses in host cells. This information also provides a theoretical basis for artificially constructing high-yield, genetically engineered cell lines for vaccines. With the development of gene editing technology in recent years, attempts have been made to genetically modify cell lines for vaccine production to better meet the need for high vaccine yield. For example, inhibition of the eIF5 A gene reduced IFN expression in MDCK cells to promote influenza virus replication, ISG knockdown in Vero cells showed a 70-fold increase in total influenza A viral particle yield [36, 37]. In this study, the total particle yield of IAV virus in USP1 knockdown cells was increased by 100-fold, which can effectively increase the yield of influenza virus and improve the efficiency of vaccine production. The next step can be to try to construct USP1 knockdown cells to support high yield of influenza virus production. Considering the complexity of intracellular signalling pathways and the co-regulation of the expression of antiviral genes by multiple gene families, multi-knockdown can also be attempted to ensure normal cell proliferation. Meanwhile, a more comprehensive understanding of the molecular mechanisms of virus-host interactions during infection is required to obtain more effective gene vaccine targets.

## Supplementary Information

Below is the link to the electronic supplementary material.

Supplementary file1 (DOCX 10.6 MB)

Supplementary file2 (DOCX 23.2 KB)

Supplementary file3 (DOCX 14.3 KB)

## Data Availability

The datasets generated during and analysed during the current study are available in the Manuscript and Supplementary Material.
