# Correction: USP1 inhibits influenza A and B virus replication in MDCK cells by mediating RIG-I deubiquitination

**DOI:** 10.1007/s00018-025-05794-7

**Published:** 2025-07-19

**Authors:** Yuejiao Liao, Siya Wang, Tian Tang, Chengfang Li, Chenhao Yang, Liyuan Ma, Jin Ye, Jiamin Wang, Di Yang, Zilin Qiao, Zhongren Ma, Zhenbin Liu

**Affiliations:** 1 Engineering Research Center of Key Technology and Industrialization of Cell-Based Vaccine, Ministry of Education, Northwest Minzu University, Lanzhou, 730030 China; 2 Gansu Tech Innovation Center of Animal Cell, Biomedical Research Center, Northwest Minzu University, Lanzhou, 730030 China; 3 Key Laboratory of Biotechnology & Bioengineering of State Ethnic Affairs Commission, Biomedical Research Center, Northwest Minzu University, Lanzhou, 730030 China; 4 Life Science and Engineering College of Northwest Minzu University, Lanzhou, 730030 China; 5 Department of Experiment & Teaching, Northwest Minzu University, Lanzhou, 730030 China

**Correction: Cellular and Molecular Life Sciences (2025) 82:200**

10.1007/s00018-025-05733-6

In this article the author’s name Chengfang Li was incorrectly written as Chengfan Li.

The original article has been corrected.

