# Supplementary material for: USP1 inhibits influenza A and B virus replication in MDCK cells by mediating RIG-I deubiquitination
Source: Cell Mol Life Sci. 2025 May 14;82(1):200. doi: 10.1007/s00018-025-05733-6 (PMC12078747; doi:10.1007/s00018-025-05733-6)
Supplement: Supplementary file 1 — Supplementary file1 (DOCX 10.6 MB) [file 18_2025_5733_MOESM1_ESM.docx]

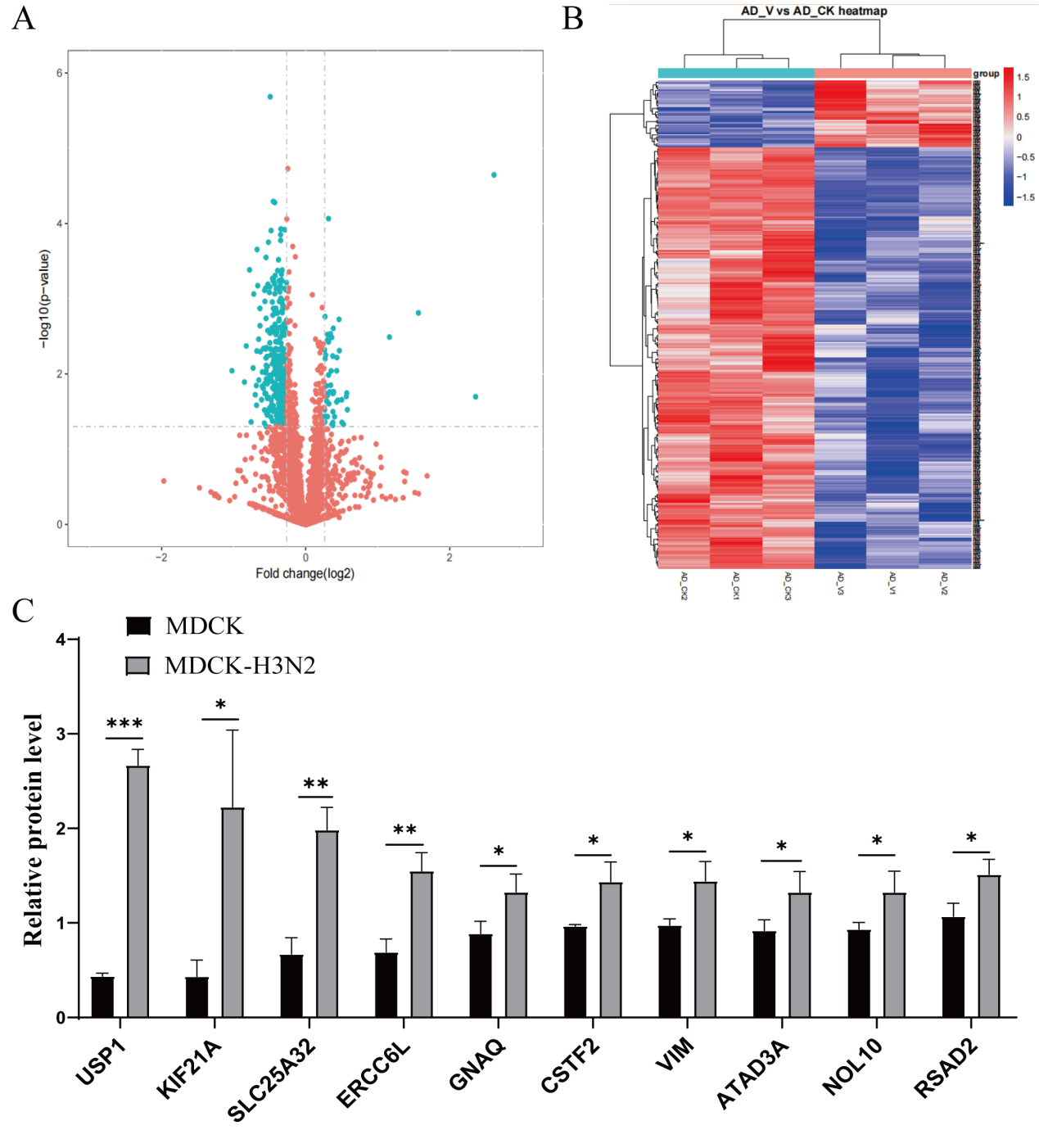


Figure S1 Different proteins are expressed in MDCK cells infected with A(H3N2). MDCK cells were infected with A(H3N2) (MOI = 1) or sham-infected with DMEM and harvested 12 hpi for analysis. (A) Volcano plots of different proteins in MDCK + H3N2 (AD_V) cells relative to uninfected MDCK (AD_CK) cells. (B) Heat map of different proteins in MDCK + H3N2 (AD_V) cells relative to uninfected MDCK (AD_CK) cells; each column represents a different sample, and each row represents a different proteins. (C) Different protein expression in H3N2 infected MDCK cells. * p < 0.05; ** p < 0.01; *** p < 0.001.


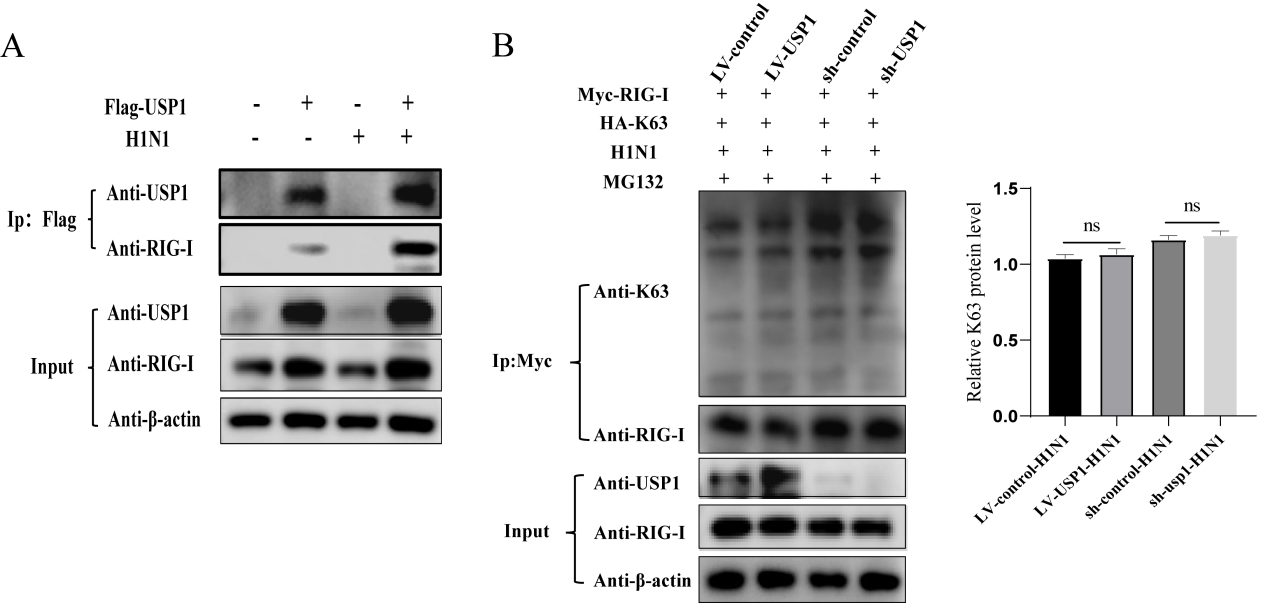


Figure S2 USP1 interacts with RIG-I does not affect the level of K63 ubiquitination of RIG-I. (A) Flag-tagged USP1 was pulled down using the Co-IP method before and after H1N1 infection and subjected to Wb analysis. (B) Myc-tagged RIG-I was pulled down using the IP method after H1N1 infection. Levels of K63 ubiquitinated RIG-1 were measured in LV-control, LV-USP1, sh-usp1, and sh-control cells using Wb. * p < 0.05; ** p < 0.01; *** p < 0.001.


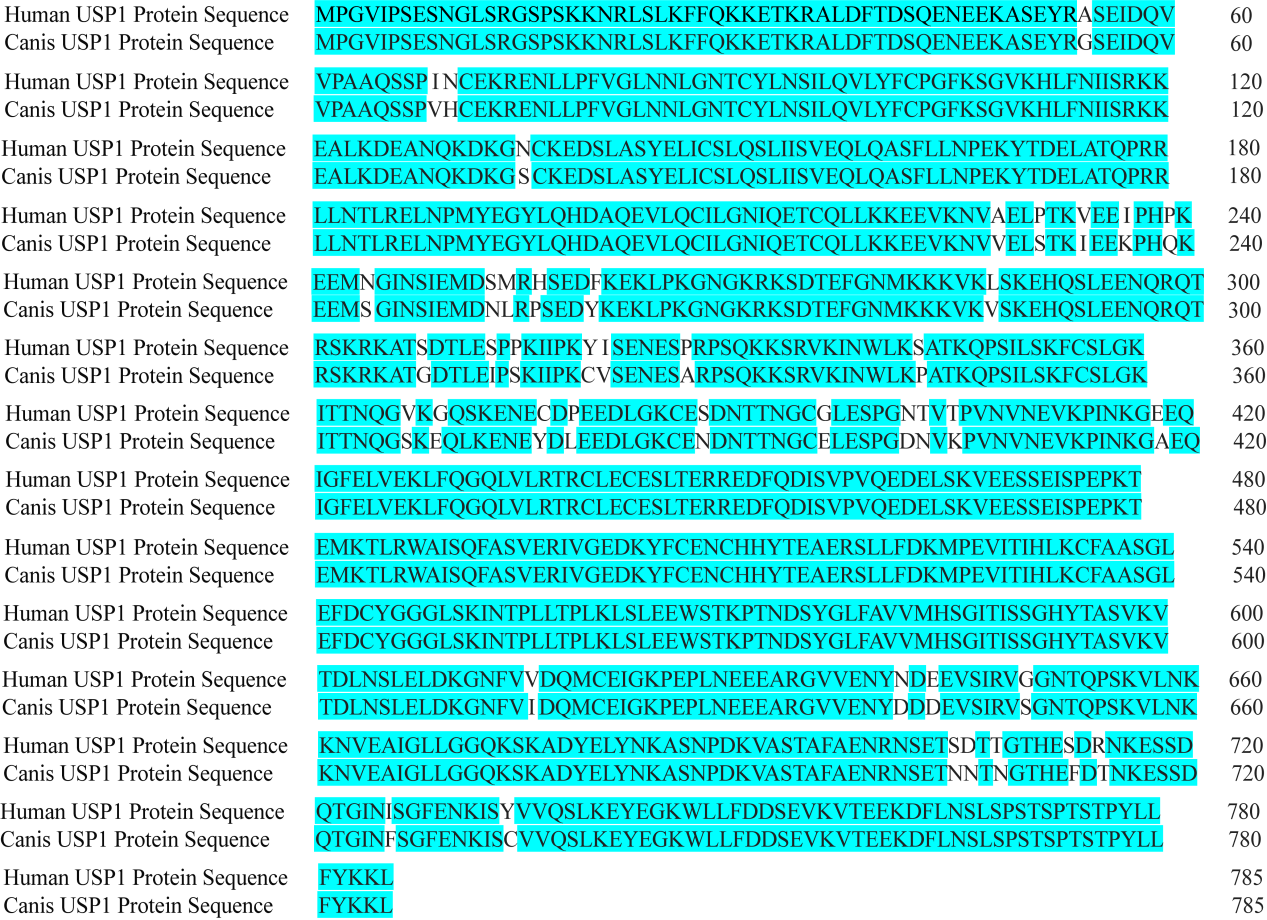


Figure S3 The sequence similarity between human USP1 and canine USP1. Identities between human-derived USP1 and canine-derived USP1 protein sequences were 741/785 (94%), positives were 758/785 (96%), gaps were 0/785 (0%). Blue:100% similarity
