# Supplementary material for: USP1 inhibits influenza A and B virus replication in MDCK cells by mediating RIG-I deubiquitination
Source: Cell Mol Life Sci. 2025 May 14;82(1):200. doi: 10.1007/s00018-025-05733-6 (PMC12078747; doi:10.1007/s00018-025-05733-6)
Supplement: Supplementary file 2 — Supplementary file2 (DOCX 23.2 KB) [file 18_2025_5733_MOESM2_ESM.docx]

**Table S1** Up-regulated of Host proteins on H1N1 viral infection.

| **Protein names** | **Sequence coverage [%]** | **Mol. weight [kDa]** | **Score** | **P.value** | **FC(2/1)** |
| --- | --- | --- | --- | --- | --- |
| USP1 | 2.1 | 60.001 | 2.9977 | 0.0000225716789796238 | 6.126881808 |
| KIF21A | 0.6 | 175.17 | 2.6936 | 0.020072978 | 5.130377598 |
| SLC25A32 | 5.1 | 35.544 | 5.7134 | 0.001543087 | 2.958933425 |
| ERCC6L | 0.9 | 143.19 | 2.8277 | 0.003233324 | 2.238711697 |
| GNAQ | 15.3 | 42.142 | 13.446 | 0.0298554 | 1.494273552 |
| CSTF2 | 21.8 | 53.778 | 68.459 | 0.0179276 | 1.485298325 |
| VIM | 83.9 | 53.597 | 323.31 | 0.019898236 | 1.478287031 |
| ATAD3A | 30.1 | 66.665 | 66.419 | 0.046830471 | 1.439849624 |
| NOL10 | 5.7 | 80.21 | 7.0946 | 0.0441542 | 1.416948976 |
| RSAD2 | 28.3 | 41.589 | 77.771 | 0.022054765 | 1.414209375 |
| HNRNPDL | 24.5 | 46.37 | 44.21 | 0.004895245 | 1.383147304 |
| SF3A2 | 14 | 48.64 | 21.611 | 0.0018829 | 1.379181981 |
| LSS | 25.1 | 82.565 | 38.726 | 0.022400137 | 1.363390789 |
| PTRH2 | 47.5 | 19.178 | 27.446 | 0.006056642 | 1.349814414 |
| PLP2 | 8.7 | 16.356 | 2.7021 | 0.009079029 | 1.347227093 |
| HIST1H1E | 42.5 | 21.865 | 66.904 | 0.023508779 | 1.346626577 |
| HIST1H2BJ | 53.2 | 13.991 | 37.449 | 0.037030146 | 1.321406994 |
| RBM12B | 5.4 | 117.21 | 8.6444 | 0.017248515 | 1.307619733 |
| HADHA | 44.8 | 81.51 | 209.04 | 0.017857186 | 1.306967819 |
| HSPE1 | 67.6 | 10.928 | 24.322 | 0.00247603 | 1.303762849 |
| RRS1 | 23.8 | 41.22 | 57.83 | 0.031715018 | 1.299347988 |
| LOC488254 | 43.8 | 13.906 | 29.364 | 0.048245429 | 1.297702154 |
| LRRC59 | 33 | 36.641 | 134.27 | 0.005786861 | 1.297524108 |
| PMPCB | 29.2 | 56.794 | 52.311 | 0.018159415 | 1.296485987 |
| HNRNPA3 | 47.4 | 39.594 | 178.28 | 0.046817848 | 1.286979081 |
| VDAC3 | 51.2 | 30.637 | 87.502 | 0.003268426 | 1.285181299 |
| HNRNPA1 | 46.1 | 38.819 | 323.31 | 0.009114787 | 1.277864039 |
| H2AFZ | 31.2 | 13.509 | 9.0283 | 0.014116285 | 1.272059324 |
| CANX | 30.4 | 67.603 | 76.393 | 0.002931563 | 1.269496987 |
| MOGS | 8.6 | 92.357 | 17.959 | 0.017312373 | 1.261413072 |
| BCL2L2-PABPN1 | 23.9 | 32.902 | 25.141 | 0.024887372 | 1.249746737 |
| ALDH5A1 | 14.5 | 47.699 | 16.635 | 0.010323164 | 1.245914888 |
| VDAC1 | 70.3 | 30.74 | 243.06 | 0.003744144 | 1.245713885 |
| MRPS25 | 20.2 | 19.967 | 12.049 | 8.60867E-05 | 1.245379402 |
| PLEC | 60.9 | 533.58 | 323.31 | 0.00358674 | 1.243392043 |
| LOC474964 | 59.3 | 37.041 | 159.13 | 0.002989038 | 1.234287656 |
| LMNA | 71.9 | 74.225 | 323.31 | 0.019352758 | 1.232512212 |
| KRT18 | 72.9 | 49.792 | 323.31 | 0.003536773 | 1.232334261 |
| LMNB2 | 20.2 | 60.621 | 23.641 | 0.022946138 | 1.224726084 |
| ATP5A1 | 47.7 | 59.674 | 323.31 | 0.014413548 | 1.221024294 |
| KRT8 | 79.5 | 54.988 | 323.31 | 0.018312398 | 1.215128879 |
| ATP5J | 40.9 | 12.858 | 15.137 | 0.005576164 | 1.212214101 |
| RPN1 | 48.6 | 68.576 | 202.96 | 0.006253642 | 1.207034975 |
| LOC483172 | 28.7 | 15.404 | 11.697 | 0.037242657 | 1.206336237 |
| NONO | 48.7 | 54.103 | 239.77 | 0.001731627 | 1.205821059 |
| SRSF1 | 54.5 | 28.329 | 63.223 | 0.036411693 | 1.204876447 |
| DLD | 29.9 | 54.153 | 54.488 | 0.043590911 | 1.203653093 |
| LOC486530 | 70.9 | 50.883 | 217.57 | 0.029902083 | 1.201558202 |
| HNRNPA2B1 | 64.6 | 37.394 | 263.04 | 0.004797289 | 1.200090355 |
