# Supplementary material for: USP1 inhibits influenza A and B virus replication in MDCK cells by mediating RIG-I deubiquitination
Source: Cell Mol Life Sci. 2025 May 14;82(1):200. doi: 10.1007/s00018-025-05733-6 (PMC12078747; doi:10.1007/s00018-025-05733-6)
Supplement: Supplementary file 3 — Supplementary file3 (DOCX 14.3 KB) [file 18_2025_5733_MOESM3_ESM.docx]

**Table S2** Putative USP1-binding proteins identified by mass spectrometry analysis.

| **Names** | **Peptides** | **Sequence coverage [%]** | **kDa** | **Participation in biological processes** |
| --- | --- | --- | --- | --- |
| HSPA8 | 25 | 43.3 | 70.897 | viral genome replication |
| FN1 | 33 | 15.8 | 282.87 | cell-matrix adhesion, cell shape |
| RIG-I(DDX58) | 10 | 14.8 | 106.05 | antiviral innate immune response |
| PRDX1 | 13 | 72.9 | 22.124 | nc |
| VCP | 11 | 21.5 | 89.302 | viral genome replication |
| PSMD11 | 8 | 22.5 | 47.463 | nc |
| PDIA3 | 7 | 17.6 | 56.75 | [cellular response to interleukin-7](https://www.ebi.ac.uk/QuickGO/term/GO:0098761) |
| ARF4 | 6 | 33.9 | 20.527 | negative regulation of apoptotic process |
| SERPINB1 | 4 | 13 | 42.817 | nc |
| PSMB2 | 3 | 16.9 | 22.781 | proteasomal protein catabolic process |
| USP10 | 3 | 6 | 89.232 | cellular response to interleukin-1 |
| YWHAB | 3 | 11.8 | 28.052 | nc |
| FTH1 | 2 | 11.5 | 21.308 | ion homeostasis |
| PSMD14 | 2 | 6.5 | 34.415 | nc |
| RHOA | 2 | 13 | 21.74 | positive regulation of NF-κB signal transduction |
| CACYBP | 1 | 6.1 | 41.385 | nc |
